# Alterations of gut microbiome and effects of probiotic therapy in patients with liver cirrhosis: A systematic review and meta-analysis

**DOI:** 10.1097/MD.0000000000032335

**Published:** 2022-12-23

**Authors:** Long Huang, Qingsheng Yu, Hui Peng, Zhou Zhen

**Affiliations:** a Department of No. 1 Surgery, The First Hospital Affiliated to Anhui University of Traditional Chinese Medicine, Hefei, Anhui Province, China; b Department of Surgery, The Second Hospital Affiliated to Anhui University of Traditional Chinese Medicine, Hefei, Anhui Province, China.

**Keywords:** gut microbiome, liver cirrhosis, meta-analysis, probiotics

## Abstract

Alterations in the gut microbiome usually occur in liver cirrhosis. Gut microbiome dysregulation damages the liver and accelerates the development of liver fibrosis. Probiotic treatment has gradually become a major method for improving the prognosis of liver cirrhosis and reducing its complications. However, alterations in the gut microbiome have revealed different results, and the therapeutic effects of various probiotics are inconsistent.

**Methods:**

We searched the PubMed, Medline, EMBASE, ScienceDirect, and Cochrane databases up to August 2022 and conducted a systematic review and meta-analysis of 17 relevant studies.

**Results:**

The counts of *Enterobacter* (standardized mean difference [SMD] −1.79, 95% confidence interval [CI]: −3.08 to −0.49) and *Enterococcus* (SMD −1.41, 95% CI: −2.26 to −0.55) increased significantly in patients with cirrhosis, while the counts of *Lactobacillus* (SMD 0.63, 95% CI: 0.12–1.15) and *Bifidobacterium* (SMD 0.44, 95% CI: 0.12–0.77) decreased significantly. Blood ammonia (weighted mean difference [WMD] 14.61, 95% CI: 7.84–21.37) and the incidence of hepatic encephalopathy (WMD 0.40, 95% CI: 0.27–0.61) were significantly decreased in the probiotic group. As for mortality (MD 0.75, 95% CI: 0.48–1.16) and the incidence of spontaneous bacterial peritonitis (WMD −0.02, 95% CI: −0.07 to 0.03), no significant differences were found between the probiotic and placebo groups.

**Conclusion:**

In summary, the gut microbiome in cirrhosis manifests as decreased counts of *Lactobacillus* and *Bifidobacterium* and increased counts of *Enterobacter* and *Enterococcus*. Targeted supplementation of probiotics in cirrhosis, including *Lactobacillus* combined with *Bifidobacterium* or *Bifidobacterium* alone, can reduce blood ammonia and the incidence of hepatic encephalopathy. The effect is similar to that of lactulose, but it has no obvious effect on mortality and spontaneous bacterial peritonitis.

## 1. Introduction

Liver cirrhosis is the terminal stage of chronic liver disease, with liver insufficiency and portal hypertension as the main manifestations. Advanced cirrhosis is often accompanied by upper gastrointestinal bleeding, infection, ascites, hepatic encephalopathy (HE), and a series of complications.^[1–3]^ HE is one of the most serious complications with diverse clinical manifestations, ranging from consciousness disorder to coma, and is associated with high mortality.^[4,5]^ Elevated ammonia levels play a vital role in the occurrence and development of HE owing to the reduced metabolic capacity of the liver.^[6]^

Gut microbiome dysregulation is linked to numerous diseases and pro-inflammatory states, such as irritable bowel syndrome, rheumatoid arthritis, systemic lupus erythematosus and liver cirrhosis.^[7,8]^ In the presence of gut microbiome dysregulation, the immune system could be erroneously directed in favor of pro-inflammatory pathways to instigate different diseases.^[9]^ In recent years, studies have suggested that gut microbiome dysregulation can occur in liver cirrhosis and is closely related to the occurrence of liver cirrhosis complications.^[10,11]^ The liver is closely connected to the intestine via the portal vein and interacts with each other.^[12]^ Gut microbiome dysregulation aggravates dysfunction of the intestinal mucosal barrier and bacterial translocation, which damages the liver and accelerates the development of liver fibrosis.^[13]^ At present, probiotics have been proven to reduce damage to hepatic cells, blood ammonia, and endotoxins, and prevent HE by regulating the imbalance of the gut microbiome and strengthening the function of the intestinal mucosa barrier.^[14–16]^ Compared with antibiotics and fecal microbial transplantation, probiotic treatment is cheaper and less harmful and has gradually become a major method for improving the prognosis of liver cirrhosis and reducing complications.^[17]^ Therefore, exploring the dysregulation characteristics of the gut microbiome in patients with liver cirrhosis will contribute to more accurate clinical diagnosis and probiotic treatment.

Liver cirrhosis is an end-stage liver disease with varying degrees of intestinal dysbiosis, intestinal barrier disorder, bacterial translocation, and inflammatory responses.^[18]^ It is essential to investigate alterations in the gut microbiome in liver cirrhosis, which play a vital role in the early diagnosis and subsequent probiotic treatment of cirrhosis.^[19]^ However, alterations in the gut microbiome have revealed different results in various studies,^[20–22]^ the therapeutic effects of various probiotics do not show consistency,^[23–25]^ and the consistency and credibility of the results need to be further explored. There is still no all‑around systematic evaluation of the therapeutic effects of various probiotics on liver cirrhosis. Therefore, we performed a meta‑analysis of a large number of randomized controlled trials (RCTs) to assess the correlation between alterations in the gut microbiome in cirrhosis and the therapeutic effects of different probiotics to provide more information about clinical probiotic treatment in cirrhosis.

## 2. Materials and Methods

### 2.1. Search strategy and selection of papers

All databases including PubMed, Medline, EMBASE, ScienceDirect, and Cochrane were searched from their inception to August 2022 using the key words: (“Gastrointestinal Microbiome” OR “gut microbiome” OR “intestinal microbiota” OR “Gastrointestinal Flora” OR “gut flora” OR “gut microbiota”OR “intestinal microbiome”) AND (“liver cirrhosis” OR “Hepatic Cirrhosis” OR “cirrhosis”), “Probiotics” AND (“liver cirrhosis” OR “Hepatic Cirrhosis” OR “cirrhosis”). All eligible studies were screened with no restrictions on country, sample size, age, sex, etc. Details of the search strategy can be found in Supplemental Digital Content, http://links.lww.com/MD/I146. The review protocol was prospectively registered in the Chinese Clinical Trial Registry.

### 2.2. Reviewing and data extraction

All included studies were required to contain either of the 2 comparisons, one of which was conducted to compare the alterations in the gut microbiome between cirrhosis and healthy controls, and the other to compare the therapeutic effects of probiotics, lactulose, and placebo groups on cirrhosis in a controlled manner. Outcomes regarding the alterations of the gut microbiome between patients with cirrhosis and healthy controls should include the counts of the gut microbiome in patients with cirrhosis and healthy controls. The outcomes in another comparison were blood ammonia, incidence rate of HE, incidence rate of spontaneous bacterial peritonitis (SBP), and morbidity. Trials that did not report any of the parameters were excluded.

General information was extracted from abstracts, including the authors, publication year, country of population, clinical characteristics of patients and outcomes, and microbiological assessment methods by 3 reviewers independently.

### 2.3. Inclusion and exclusion criteria

Stricter inclusion criteria were required for this meta-analysis: the study should contain either of the 2 comparisons: one was conducted to compare the alterations of the gut microbiome between cirrhosis and healthy controls, and the other was to compare the therapeutic effects of probiotics, lactulose, and placebo groups on cirrhosis; the study provided at least one of the outcomes, and the outcome could be extracted; if 2 studies were from the same institution or the same author, the study content and the included patients should be different; and the studies were full-text availability.

The exclusion criteria were as follows: studies that did not report any of the data or the data could not be extracted in the required form; studies of comments, animal models, conferences, and reviews; and overlaps between authors or institutions in the studies.

### 2.4. Quality of studies

Two reviewers completed the quality assessment using the Newcastle-Ottawa scale for cohort studies or the Jadad score for RCTs to evaluate all included studies.^[26,27]^ All papers were assessed for the risk of bias as suggested in the Cochrane Handbook.^[28]^

### 2.5. Data analysis

The verified data were analyzed using Review Manager (Version 5.3. Copenhagen: The Nordic Cochrane Centre, The Cochrane Collaboration, 2014.). The odds ratio, mean difference (MD), and corresponding 95% confidence intervals (CIs) were calculated for dichotomous and continuous outcome data. The weighted mean difference (WMD) was used if the measurement units of the data were the same. If the measurement units of the data were different, a standardized mean difference (SMD) was calculated, including alterations in various gut microbiomes. Statistical heterogeneity was assessed using the *I*^2^ test and *Q* tests to verify the accuracy of the fixed or random effects model. A significant effect was assumed if the 95% CI did not include a value 1.0 for odds ratio or 0 for MD. A fixed-effect model was used in cases where there was no relevant statistical heterogeneity when *I*^2^ was <50% and *P* > .1. A random-effect model was used when *I*^2^ was >50% and *P* was <.1. If the heterogeneity was high (*I*^2^ > 50% and *P* < .1), subgroup analysis could be used to decrease the risk of bias. Subgroup analysis was conducted to decrease heterogeneity and observe the results between subgroups. Funnel plots were used to evaluate the publication bias.

## 3. Results

### 3.1. Description of included studies

**Figure 1.**
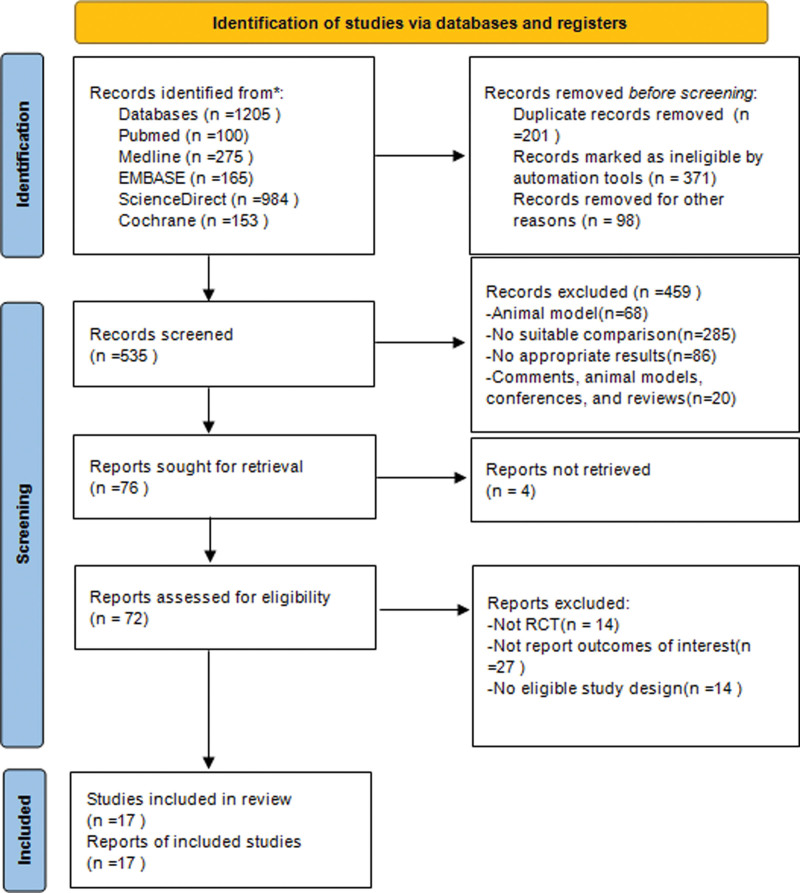
Flow chart of the search strategy and study selection progress.

In total, 535 relevant trials were identified from the online database after an initial literature search was conducted. After carefully checking titles and abstracts according to our predefined inclusion and exclusion criteria, 76 references were retained for further evaluation. We then evaluated the remaining 76 articles; 52 were excluded for eligibility, and 7 were excluded because of duplication. Eventually, 4 studies^[29–32]^ concerning the gut microbiome between cirrhosis and healthy controls and 13 RCTs^[33–45]^ concerning the treatments of cirrhosis among probiotics, lactulose, and placebo were included in this meta-analysis (Fig. 1).

The basic characteristics of the included 17 studies are summarized in Table 1. The evaluation of quality and risk of bias in the included 17 studies was performed using the Newcastle-Ottawa scale for cohort studies or the Jadad score for RCTs, according to the bias risk assessment method provided by the Cochrane Handbook.

**Table 1:** Clinical characteristics and microbiology assessment of included studies.

Study	Country	Type	Group	Probiotics	No. of patients	Age	Course of treatment	Score
Zhao et al 2004	China	RCT	Healthy control vs cirrhosis	None	20:50	49.8 (25–70):50.5 (14–75)	NA	7
Lu et al 2011	China	Cohort study	Healthy control vs cirrhosis	None	32:31	43.1 ± 5.2:49.0 ± 4.8	NA	7
Liu et al 2012	China	Cohort study	Healthy control vs cirrhosis	None	4:6	40–60	NA	5
MOU et al 2018	China	Cohort study	Healthy control vs cirrhosis	None	40:52	36.7 ± 10.1:53.1 ± 11.4	NA	7
Loguercio et al 1995	Italy	RCT	Probiotics vs lactulose	*Enterococcus*	14:11	58.1 (42–76):58.8 (41–71)	4 wk	4
Liu et al 2004	China	RCT	Probiotics vs placebo	*Lactobacillus* + *Streptococcus*	20:15	55 ± 12:57 ± 12	30 d	6
Malaguarnera et al 2007	Italy	RCT	Probiotics vs placebo	*Bifidobacterium*	30:30	46 ± 11:45 ± 12	120 d	6
Bajaj et al 2008	USA	RCT	Probiotics vs placebo	Yogurt	14:6	52 ± 8:54 ± 4	60 d	4
Sharma et al 2008	India	RCT	Probiotics vs lactulose	*Streptococcus* + *Clostridium* + *Lactobacillus*	31:31	43.5 ± 12.1:39.5 ± 13.0	1 mo	4
Malaguarnera et al 2010	Italy	RCT	Probiotics vs lactulose	*Bifidobacterium*	63:62	NA:50.1 ± 9.4	60 d	4
Pereg et al 2011	Israel	RCT	Probiotics vs placebo	*Lactobacillus* + *Bifidobacterium* + *Streptococcus*	18:18	63.2 ± 10.5:65.9 ± 8.4	6 mo	6
Mittal et al 2011	India	RCT	Probiotics vs lactulose vs placebo	Unknown	34:35:31	44.25 (11.8):43.85 (10.9):41.20 (11.9)	3 mo	4
Agrawal et al 2012	India	RCT	Probiotics vs lactulose vs placebo	*Lactobacillus* + *Bifidobacterium* + *Streptococcus*	64:68:65	45.4 ± 11.7:41.7 ± 10.7:46.0 ± 11.2	3 mo	4
Pande et al 2012	India	RCT	Probiotics vs placebo	*Enterobacter*	55:55	43 (16–72):46 (16–75)	24 wk	6
Bajaj et al 2014	USA	RCT	Probiotics vs placebo	*Lactobacillus*	14:16	58.4 ± 3.8:58.5 ± 4.5	8 wk	5
Lunia et al 2014	India	RCT	Probiotics vs placebo	*Lactobacillus* + *Bifidobacterium* + *Streptococcus*	76:62	48.5 ± 10.5:49.4 ± 11.5	3 mo	4
Dhiman et al 2014	India	RCT	Probiotics vs placebo	*Lactobacillus* + *Bifidobacterium* + *Streptococcus*	66:64	48.0 (45.2–50.8):50.1 (47.6–52.5)	6 mo	5

NA = not available, RCT = randomized controlled trial.

In terms of the detection method of the gut microbiome, the gut microbiome in 4 studies was analyzed using high-throughput sequencing of the 16S ribosomal ribonucleic acid gene, real-time polymerase chain reaction, and the VITEK automatic microbiological identification system. Comparisons of the gut microbiomes in the 4 studies were matched for *Enterococcus*, *Enterobacter*, *Bifidobacterium*, *Lactobacillus*, *Bacteroides*, and *Clostridium*. Comparisons of probiotics, lactulose, and placebo in the 13 studies were matched for serum ammonia level, incidence of HE development, SBP, and mortality.

### 3.2. Gut microbiome

#### 3.2.1. *Enterobacter*.

**Figure 2.**
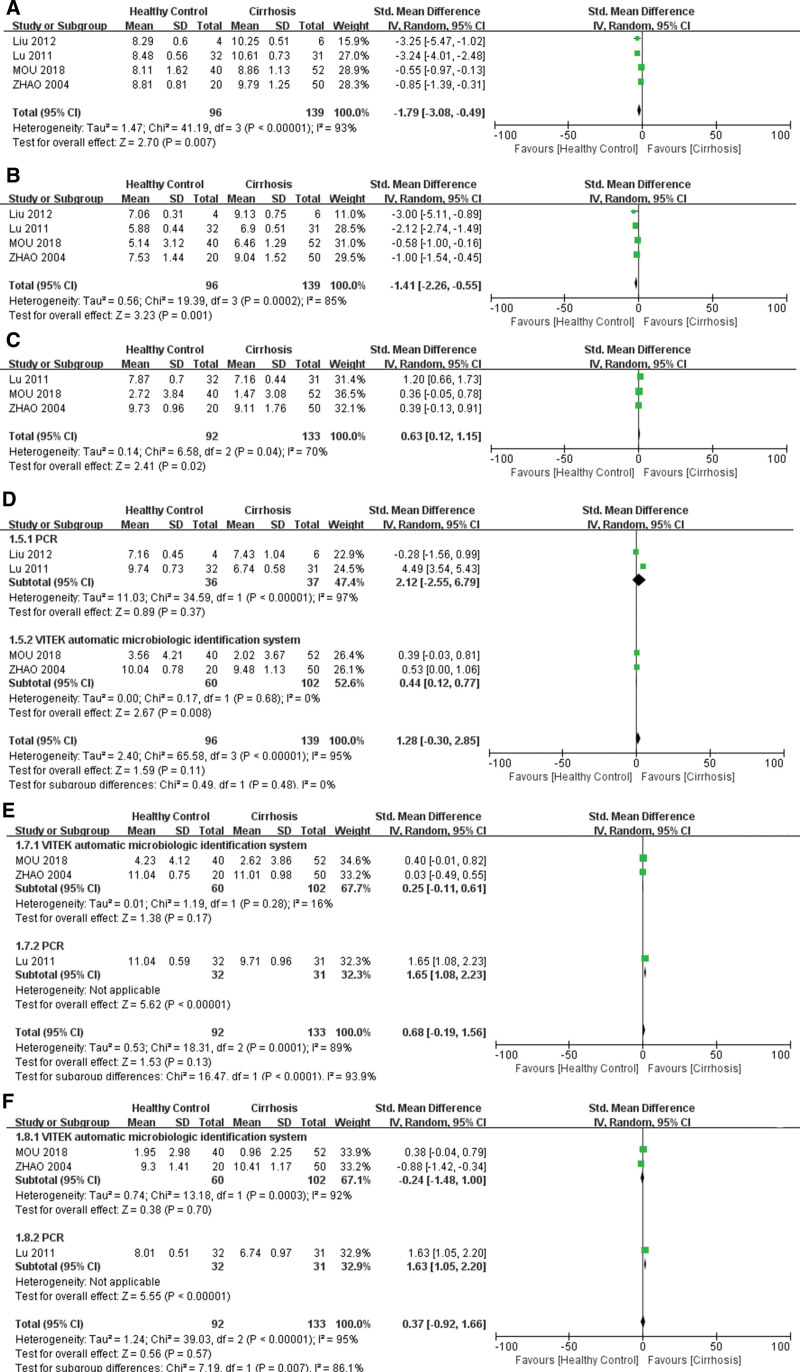
Forest plot of the gut microbiome between cirrhosis and healthy controls. (A) *Enterobacter*. (B) *Enterococcus*. (C) *Lactobacillus*. (D) *Bifidobacterium*. (E) *Bacteroidetes*. (F) *Clostridium*.

Four studies compared *Enterobacter* counts between healthy controls and patients with cirrhosis. The included studies revealed heterogeneity (*χ*^2^ = 41.19, df = 3, *P* < .00001, *I*^2^ = 93%). The outcome showed that the counts of *Enterobacter* increased significantly in cirrhosis (*Z* = 2.70, *P* = .007, SMD −1.79, 95% CI: −3.08 to −0.49) in a random-effects meta-analysis (Fig. 2A).

#### 3.2.2. *Enterococcus*.

The 4 included studies showed heterogeneity (*χ*^2^ = 19.39, df = 3, *P* = .0002, *I*^2^ = 85%). In our research, the count of *Enterococcus* was significantly increased in the cirrhosis group (*Z* = 3.23, *P* = .001, SMD −1.41, 95% CI: −2.26 to −0.55) in a random-effects meta-analysis (Fig. 2B).

#### 3.2.3. *Lactobacillus*.

Three studies compared the *Lactobacillus* counts between healthy controls and patients with cirrhosis. Heterogeneity was observed among the included studies (*χ*^2^ = 6.58, df = 2, *P* = .04, *I*^2^ = 70%). The results demonstrated that the *Lactobacillus* count was significantly decreased in patients with cirrhosis (*Z* = 6.58, *P* = .02, SMD 0.63, 95% CI: 0.12–1.15) in a random-effects meta-analysis (Fig. 2C).

#### 3.2.4. *Bifidobacterium*.

Four studies compared *Bifidobacterium* levels between patients with cirrhosis and healthy controls. We conducted subgroup analysis of the detection method of the gut microbiome for heterogeneity (*χ*^2^ = 65.58, df = 3, *P* < .00001, *I*^2^ = 95%). The *Bifidobacterium* counts were not significantly different between patients with cirrhosis and healthy controls (*Z* = 1.59, *P* = .11, SMD 1.28, 95% CI: −0.30 to 2.85). However, we found decreased counts of *Bifidobacterium* in cirrhosis using the VITEK automatic microbiological identification system (*Z* = 2.67, *P* = .008, SMD 0.44, 95% CI: 0.12–0.77) in the subgroup analysis (Fig. 2D).

#### 3.2.5. *Bacteroidetes*.

Three studies have reported alterations in *Bacteroidetes* in patients with cirrhosis. Subgroup analysis of the detection method of the gut microbiome was performed for heterogeneity (*χ*^2^ = 18.31, df = 2, *P* = .0001, *I*^2^ = 89%). Although subgroup analysis revealed significant differences (*Z* = 5.62, *P* < .00001, SMD 1.65, 95% CI: 1.08– 2.23), a random-effects meta-analysis model showed a similar result (*Z* = 1.53, *P* = .13, SMD 0.68, 95% CI: −0.19 to 1.56) between healthy controls and patients with cirrhosis (Fig. 2E).

#### 3.2.6. *Clostridium*.

Sub-group analysis was performed for heterogeneity (*χ*^2^ = 39.03, df = 2, *P* < .00001, *I*^2^ = 95%). Although the subgroup analysis revealed a significant difference (*Z* = 5.55, *P* < .00001, SMD 1.63, 95% CI: 1.05–2.20), the counts of *Clostridium* revealed no significant difference between cirrhosis and healthy control (*Z* = 0.56, *P* = .57, SMD 0.37, 95% CI: −0.92 to 1.66) (Fig. 2F).

### 3.3. Comparison among probiotics, lactulose, and placebo groups

#### 3.3.1. Ammonia.

**Figure 3.**
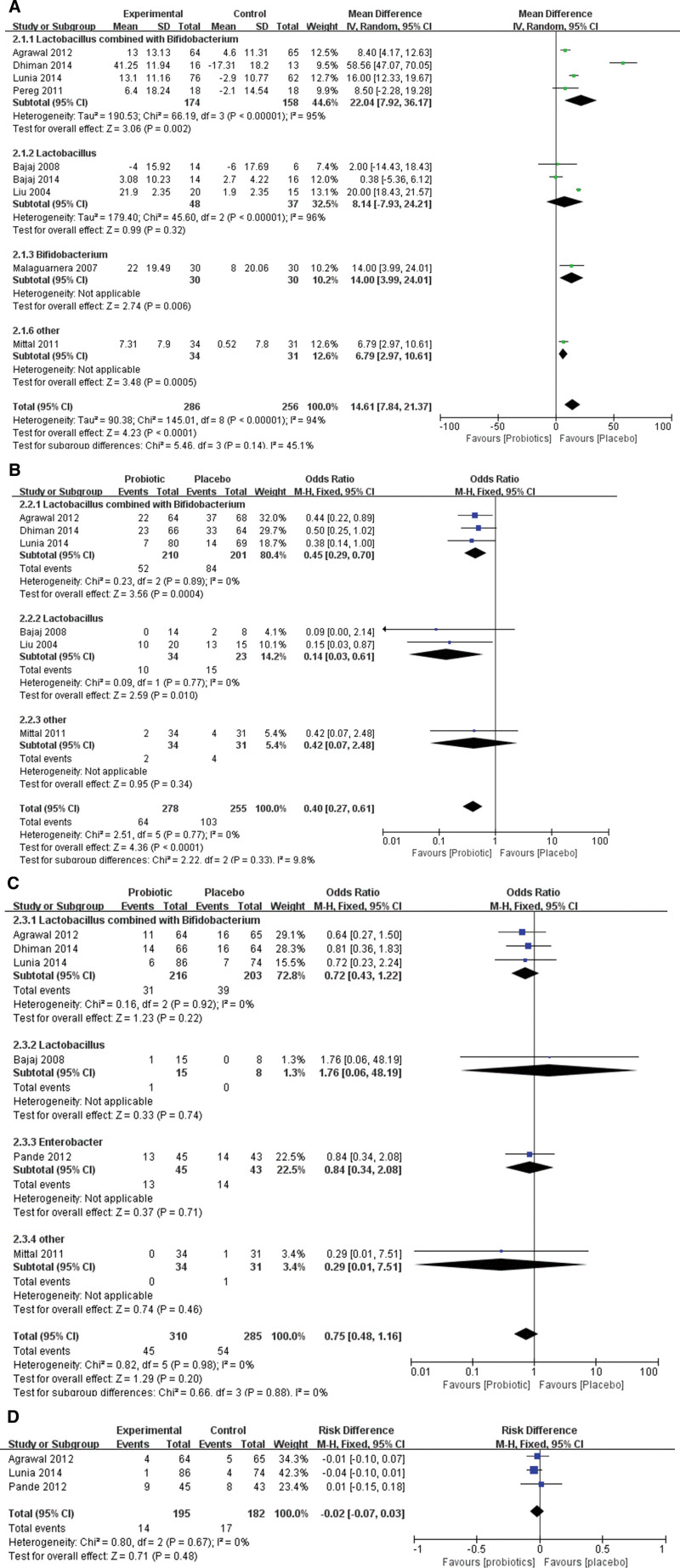
Forest plot displaying the comparison between cirrhosis and placebo groups. (A) Comparison of blood ammonia. (B) Comparison of the incidence of hepatic encephalopathy. (C) Comparison of mortality. (D) Comparison of spontaneous bacterial peritonitis.

In our meta-analysis, 9 studies reported changes in blood ammonia between the probiotics group and the placebo group, and there was statistical heterogeneity among the groups (*χ*^2^ = 145.01, df = 8, *P* < .00001, *I*^2^ = 94%). A random-effects meta-analysis model showed that probiotics could significantly decrease blood ammonia compared with the placebo group (*Z* = 4.23, *P* < .0001, WMD 14.61, 95% CI: 7.84–21.37). A subgroup analysis which was conducted for various probiotics demonstrated that *Lactobacillus* combined with *Bifidobacterium* (*Z* = 3.06, *P* = .002, WMD 22.04, 95% CI: 7.92–36.17), and *Bifidobacterium* alone (*Z* = 2.74, *P* = .006, WMD 14.00, 95% CI: 3.99–24.01) could decrease blood ammonia significantly (Fig. 3A).

**Figure 4.**
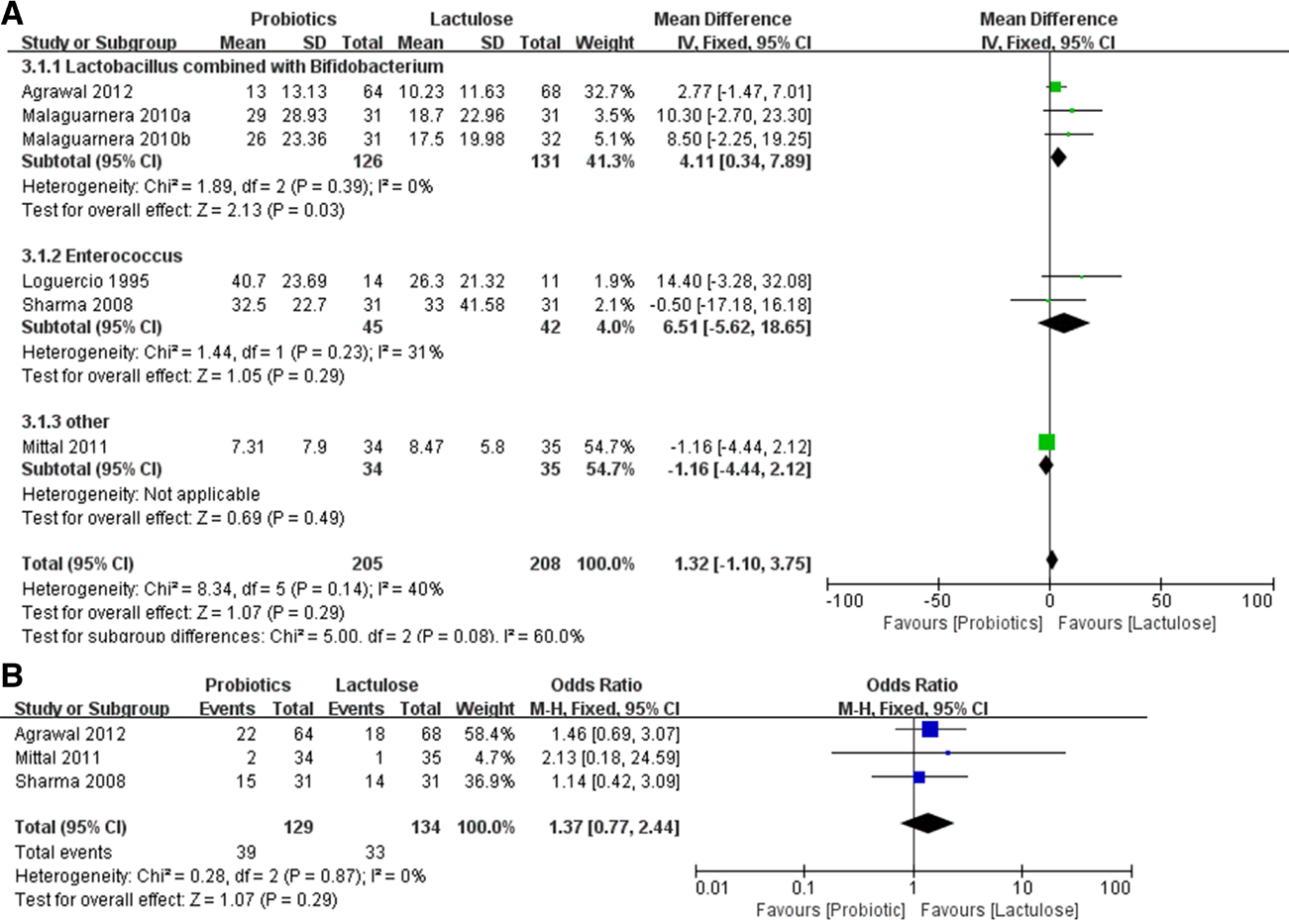
Forest plot displaying the comparison between cirrhosis and lactulose groups. (A) Comparison of blood ammonia. (B) Comparison of the incidence of hepatic encephalopathy.

Six RCTs analyzed the effect of probiotics and lactulose on changes in blood ammonia in patients with cirrhosis, and there was no statistical heterogeneity among the groups (*χ*^2^ = 8.34, df = 5, *P* = .14, *I*^2^ = 40%). A fixed-effects meta-analysis model showed that probiotics and lactulose had similar effects on the decrease in blood ammonia levels (*Z* = 1.07, *P* = .29, WMD 1.32, 95% CI: −1.10 to 3.75). However, subgroup analysis revealed that *Lactobacillus* combined with *Bifidobacterium* could significantly decrease blood ammonia compared with the lactulose group (*Z* = 2.13, *P* = .03, WMD 4.11, 95% CI: 0.34–7.89) (Fig. 4A).

#### 3.3.2. The incidence of HE.

In our meta-analysis, 6 studies reported the incidence of HE between the probiotic and placebo groups, and there was no statistical heterogeneity among the groups (*χ*^2^ = 2.51, df = 5, *P* = .77, *I*^2^ = 0%). A fixed-effects meta-analysis model showed that probiotics could significantly decrease the incidence of HE compared to the placebo group (*Z* = 4.36, *P* < .0001, WMD 0.40, 95% CI: 0.27–0.61). A subgroup analysis, which was conducted for various probiotics, demonstrated that *Lactobacillus* combined with *Bifidobacterium* (*Z* = 3.56, *P* = .0004, WMD 0.45, 95% CI: 0.29–0.70), and *Lactobacillus* alone (*Z* = 2.56, *P* = .010, WMD 0.14, 95% CI: 0.03–0.61) could decrease the incidence of HE significantly compared with placebo group (Fig. 3B).

Only 3 RCTs reported the effect of probiotics and lactulose on the incidence of HE in patients with cirrhosis, and there was no statistical heterogeneity among the groups (*χ*^2^ = 0.28, df = 2, *P* = .87, *I*^2^ = 0%). A fixed-effects meta-analysis model showed that probiotics and lactulose had similar effects in decreasing the incidence of HE (*Z* = 1.07, *P* = .29, WMD 1.37, 95% CI: 0.77–2.44) (Fig. 4B).

### 3.4. Mortality

In our meta-analysis, 6 studies reported mortality between the probiotic and placebo groups, and there was no statistical heterogeneity among the groups (*χ*^2^ = 0.82, df = 5, *P* = .98, *I*^2^ = 0%). A fixed-effects meta-analysis model revealed that the probiotic group had similar effects on mortality as the placebo group (*Z* = 1.29, *P* = .20, WMD 0.75, 95% CI: 0.48–1.16). Although subgroup analysis was conducted, *Lactobacillus* combined with *Bifidobacterium* (*Z* = 1.23, *P* = .22, WMD 0.72, 95% CI: 0.43–1.22), *Lactobacillus* alone (*Z* = 0.33, *P* = .74, WMD 1.76, 95% CI: 0.06–48.19), and *Enterobacter* alone (*Z* = 0.37, *P* = .71, WMD 0.84, 95% CI: 0.34–2.08) all revealed no significant difference when compared with the placebo group (Fig. 3C).

### 3.5. SBP

Only 3 RCTs reported the effect of probiotics on the incidence of SBP in cirrhotic patients, and there was no statistical heterogeneity between the probiotic and placebo groups (*χ*^2^ = 0.80, df = 2, *P* = .67, *I*^2^ = 0%). A fixed-effects meta-analysis model showed that probiotics had a similar incidence of SBP when compared to the placebo group (*Z* = 0.71, *P* = .48, WMD −0.02, 95% CI: −0.07 to 0.03) (Fig. 3D).

### 3.6. Publication bias

**Figure 5.**
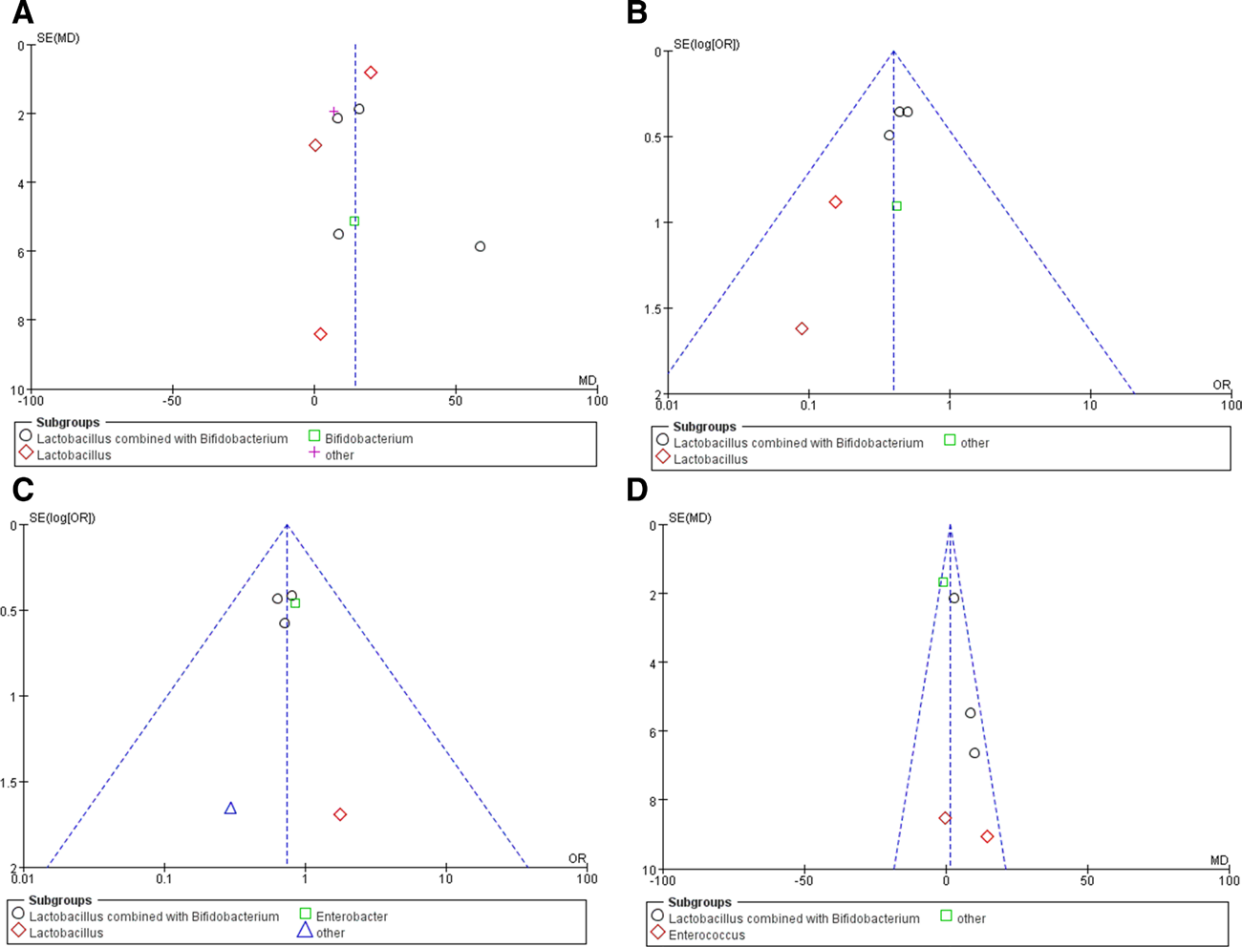
Publication bias in the funnel plots. (A) Publication bias in the changes of blood ammonia between the probiotics and the placebo groups. (B) Publication bias in the incidence of HE. (C) Publication bias mortality. (D) Publication bias the changes of blood ammonia between the probiotics and the lactulose groups. HE = hepatic encephalopathy.

Funnel plots suggested that publication bias was found in the changes in blood ammonia between the probiotics and placebo groups, as the funnel plots showed asymmetry (Fig. 5A). Funnel plots suggested that no publication bias was found in the incidence of HE (Fig. 5B), mortality (Fig. 5C), and the changes in blood ammonia between the probiotics and lactulose groups (Fig. 5D) for the funnel plots showed good symmetry.

## 4. Discussion

Dysbiosis of the gut microbiome usually occurs in patients with liver cirrhosis as a result of pathological interactions between the liver and intestine. Some studies have accurately evaluated the common changes in the composition of the gut microbiome in patients with liver cirrhosis, including the reduction in beneficial bacteria and the increase in potentially pathogenic bacteria.^[46]^ Therefore, it plays a vital role in investigating the characteristics of gut microbiome changes in patients with cirrhosis and exploring probiotic treatments for clinical cirrhosis. The results of this meta-analysis revealed that cirrhotic patients had different degrees of gut microbiome disorder, which was specifically manifested by decreased counts of *Lactobacillus* and *Bifidobacterium* and significantly increased counts of *Enterobacter* and *Enterococcus*. Probiotics including *Lactobacillus* combined with *Bifidobacterium* and *Bifidobacterium* alone could effectively reduce blood ammonia and the incidence of HE in patients with liver cirrhosis, and the effect was similar to that of lactulose, but it had no obvious effect on mortality.

Previous studies have demonstrated that intestinal flora disorder is a vital risk factor for severe complications, such as HE and spontaneous peritonitis in patients with liver cirrhosis.^[47]^ In recent years, probiotics have attracted extensive attention in the treatment of liver cirrhosis because of their low cost and minimal adverse reactions. However, previous studies regarding probiotics showed limitations such as a small sample size, lack of evidence-based medical evidence, and incomplete evaluation indicators.^[48]^ Therefore, our meta-analysis study mainly focused on the systematic evaluation of the efficacy of various probiotics in the treatment of liver cirrhosis, and discussed the relationship between the alterations of the gut microbiome in cirrhosis and the effects of probiotics in preventing complications and reducing mortality.

The systemic inflammatory reaction, portal venous blood stasis, and oxidative stress, which gradually arise in cirrhotic patients with portal hypertension, further damage the barrier function of the gut and change its permeability, leading to bacterial translocation.^[49]^ Impaired intestinal mucosal barrier function may lead to qualitative and quantitative changes in the gut microbiome associated with end-stage liver disease, which can be clarified using new sequencing techniques.^[50]^ In addition, some vital factors including overgrowth of intestinal bacteria and changes in intestinal permeability may affect cirrhosis-related complications, especially HE.^[51]^ Therefore, the dysbiosis pattern of the gut microbiome may serve as a reasonable biomarker for the diagnosis and prognosis of cirrhosis.^[52]^

The degree of liver injury is closely associated with intestinal dysbiosis severity. Microorganisms and their metabolites can reach the liver easily by increasing intestinal permeability and bacterial translocation, which could affect bile acid metabolism and systemic inflammation, and further aggravate intestinal dysregulation.^[53]^ Ammonia and endotoxins easily enter the blood circulation and directly lead to liver damage when *Enterobacteriaceae* are high and anaerobic bacteria, including *bifidobacteria*, are low. Probiotics are microbial preparations that are beneficial to the intestine. Probiotic therapy improves intestinal dysbiosis and bacterial translocation, and improves the prognosis of liver cirrhosis.^[54]^ Probiotics can inhibit the growth of pathogenic bacteria by acidifying the gut lumen, competing for nutrients, and producing antibacterial substances, thereby improving the prognosis of patients with liver cirrhosis. Studies have shown that probiotics can reduce the ammonia level in the blood and the pH value in the intestine, thereby reducing intestinal permeability, inflammatory reactions, oxidative stress in hepatocytes, and improving the ability of the liver to remove blood ammonia.^[55]^ Probiotics produce many inactive metabolic byproducts, such as bacteriocins, organic acids, acetaldehyde, diacetyl, ethanol, and hydrogen peroxide.^[56]^ Bacteriocins inhibit pathogenic microorganisms; therefore, probiotics can be used to prevent and treat infections. The therapeutic mechanisms of probiotics in liver cirrhosis may be as follows: prevention of infection, improvement of hemodynamic disorders of liver cirrhosis, prevention of HE, and improvement of liver function. However, the heterogeneity of probiotic or probiotic combinations provides evidence for the effectiveness of probiotics in liver cirrhosis.

*Bifidobacterium* belongs to *Actinobacteria*, which is the main microbiota in healthy breastfed infants, and its levels remains relatively stable during adulthood and tends to decrease with age. *Lactobacillus* is also common in healthy intestines and is often combined with *bifidobacteria* as probiotics to treat diseases. In our study, *Bifidobacterium* and *Lactobacillus* combined or applied alone in the treatment of liver cirrhosis achieved satisfactory outcomes, which could reduce the incidence of blood ammonia and HE.

*Clostridium butyricum*, a gram-positive anaerobic bacillus, produces short chain fatty acids (SCFA), mainly butyrate and acetate, by fermenting undigested carbohydrates.^[57]^ SCFA can change the intestinal flora and restore intestinal barrier function by transferring them from the intestine to the liver through the portal vein. Therefore, ammonia, endotoxin, and inflammatory cytokine tumor necrosis factor-α in the portal vein can be effectively reduced. However, *Clostridium* showed no significant difference between patients with cirrhosis and healthy controls in this meta-analysis. In a meta-analysis of probiotics, only 1 study reported the effects of *Clostridium* combined with other probiotics in the treatment of liver cirrhosis. Therefore, more RCTs are needed to explore the effect of *Clostridium* in the treatment of liver cirrhosis.

This meta-analysis revealed that *Enterobacteriaceae* and *Enterococcus* increased in cirrhotic patients, which may be related to impairment of the intestinal mucosal barrier function caused by cirrhosis. Cirrhotic patients often present with increased intestinal permeability, which is caused by elevated endotoxins produced by increased *Enterobacteriaceae* in the intestine. Meanwhile, endotoxins can aggravate the manifestations of liver cirrhosis and lead to complex complications related to cirrhosis, such as HE. *Enterococcus* strains produce various bacteriocins called Enterococcins. Although Enterococcins, which are produced by strains of the genus *Enterococcus*, can inhibit closely related species and gram-positive pathogens, and *Enterococcus*-related strains (such as vancomycin-resistant *Enterococcus*, vancomycin-resistant enterococcus) may spread multiple drug resistance and virulence genes, which could cause safety issues.^[58]^ In this analysis, only 1 study reported the treatment of liver cirrhosis using *Enterococcus* and *Enterobacter* as probiotics alone. Therefore, the results regarding *Enterococcus* and *Enterobacter* were unconvincing, and *Enterococcus* and *Enterobacter* alone as probiotics to treat liver cirrhosis required further evidence-based medical support.

Probiotics are a promising field for the treatment of liver cirrhosis, but some problems still require further research. First, probiotics can be an important part of the treatment of various diseases, but 1 scheme cannot be applied to all diseases; therefore, we need to select the optimal probiotic or the best combination of probiotics through more rigorous research for the treatment of liver cirrhosis and its complications in the future. Second, although probiotics could improve the clinical outcome of liver cirrhosis, there are still certain safety risks associated with the application of probiotics. However, there seems to be a lack of systematic reporting of adverse events in all probiotic trials thus far.^[59]^ Moreover, anecdotal reports indicate that probiotics may worsen outcomes, particularly in patients receiving radiotherapy.^[60]^ Third, probiotic products, such as SCFA and bacteriocins, can replace probiotics in some cases. Therefore, the application of these metabolites may have probiotic effects without the corresponding risks.

Although 26 high-quality documents were included in this study, there were still a few limitations: the source of cirrhosis should be considered because intestinal biodiversity varies with geographical sources. Most of the data in our study were obtained from Chinese patients, which means that the conclusions based on our results cannot be directly applied to all races; some of the analyses in this study showed a high heterogeneity; different measurement methods resulted in different bacterial count units, which led to heterogeneity; the etiology of cirrhosis was not classified; and the gut microbiome in the vast majority of research has been primarily studied using stool bacterial communities as a proxy. However, some bacterial communities from the small intestine and those embedded within the intestinal mucosa have been neglected.^[61]^ To further evaluate the efficacy of various probiotics, a comprehensive meta-analysis regarding the different etiologies of cirrhosis in the same region is needed and posttreatment events should be clearly recorded.

In summary, cirrhotic patients show different degrees of gut microbiome disorder, which is specifically manifested by decreased counts of *Lactobacillus* and *Bifidobacterium*, and significantly increased counts of *Enterobacter* and *Enterococcus*. Targeted supplementation of probiotics, including *Lactobacillus* combined with *Bifidobacterium* and *Bifidobacterium* alone, can reduce blood ammonia and the incidence of HE in patients with liver cirrhosis. The effect is similar to that of lactulose, but it has no obvious effect on mortality and SBP.

## Acknowledgments

We would like to acknowledge the support of the Department of No. 1 Surgery, The First Hospital Affiliated to Anhui Chinese Medical University, China.

## Author contributions

**Conceptualization:** Long Huang.

**Data curation:** Long Huang, Hui Peng, Zhou Zhen.

**Formal analysis:** Hui Peng, Zhou Zhen.

**Funding acquisition:** Long Huang, Qingsheng Yu.

**Investigation:** Long Huang.

**Methodology:** Long Huang, Hui Peng, Zhou Zhen.

**Project administration:** Hui Peng, Zhou Zhen.

**Resources:** Qingsheng Yu.

**Software:** Long Huang, Hui Peng, Zhou Zhen.

**Supervision:** Zhou Zhen.

**Validation:** Long Huang, Qingsheng Yu.

**Visualization:** Long Huang, Hui Peng, Zhou Zhen.

**Writing – original draft:** Long Huang.

**Writing – review & editing:** Long Huang.

## Supplementary Material
