# Supplementary material for: Alterations of gut microbiome and effects of probiotic therapy in patients with liver cirrhosis: A systematic review and meta-analysis
Source: Medicine (Baltimore). 2022 Dec 23;101(51):e32335. doi: 10.1097/MD.0000000000032335 (PMC9794299; doi:10.1097/MD.0000000000032335)
Supplement: Supplementary file 1 [file medi-101-e32335-s001.pdf]

Supplemental Table 1 Search strategy in meta-analysis

**ID**	**Search**
#1	Keyword of (Gastrointestinal Microbiome OR gut microbiome OR intestinal microbiota OR Gastrointestinal Flora OR gut flora OR gut microbiota OR intestinal microbiome)
#2	Keyword of (Probiotics)
#3	Keyword of (liver cirrhosis OR Hepatic Cirrhosis OR cirrhosis)
#4	(#1 AND #3), (#2 AND #3)

Last update search: August 2022.
